# Baseline Cerebrospinal Fluid α-Synuclein in Parkinson’s Disease Is Associated with Disease Progression and Cognitive Decline

**DOI:** 10.3390/diagnostics12051259

**Published:** 2022-05-18

**Authors:** Anna Emdina, Peter Hermann, Daniela Varges, Sabine Nuhn, Stefan Goebel, Timothy Bunck, Fabian Maass, Matthias Schmitz, Franc Llorens, Niels Kruse, Paul Lingor, Brit Mollenhauer, Inga Zerr

**Affiliations:** 1 Department of Neurology, University Medical Center Göttingen, 37075 Göttingen, Germany; anna.emdina@stud.uni-goettingen.de (A.E.); d.varges@med.uni-goettingen.de (D.V.); sabine.nuhn@med.uni-goettingen.de (S.N.); stefan.goebel@med.uni-goettingen.de (S.G.); timothy.bunck@med.uni-goettingen.de (T.B.); fabian.maass@med.uni-goettingen.de (F.M.); matthias.schmitz@med.uni-goettingen.de (M.S.); franc.llorens@gmail.com (F.L.); brit.mollenhauer@med.uni-goettingen.de (B.M.); ingazerr@med.uni-goettingen.de (I.Z.); 2 Bellvitge Biomedical Research Institute (IDIBELL), Hospitalet de Llobregat, 08908 Barcelona, Spain; 3 Network Center for Biomedical Research in Neurodegenerative Diseases (CIBERNED), Instituto de Salud Carlos III, 28031 Madrid, Spain; 4 Department of Neuropathology, University Medical Centre Göttingen, 37075 Göttingen, Germany; n.kruse@med.uni-goettingen.de; 5 Department of Neurology, Klinikum Rechts der Isar, Technical University of Munich, 80333 Munich, Germany; paul.lingor@tum.de; 6 Paracelsus-Elena-Klinik, 34128 Kassel, Germany; 7 German Center for Neurodegenerative Diseases (DZNE), 37075 Göttingen, Germany

**Keywords:** Parkinson’s disease, dementia, dementia with Lewy bodies, alpha-synuclein, biomarker, cerebrospinal fluid

## Abstract

Biomarkers are increasingly recognized as tools in the diagnosis and prognosis of neurodegenerative diseases. No fluid biomarker for Parkinson’s disease (PD) has been established to date, but α-synuclein, a major component of Lewy bodies in PD and dementia with Lewy bodies (DLB), has become a promising candidate. Here, we investigated CSF α-synuclein in patients with PD (*n* = 28), PDD (*n* = 8), and DLB (*n* = 5), applying an electrochemiluminescence immunoassay. Median values were non-significantly (*p* = 0.430) higher in patients with PDD and DLB (287 pg/mL) than in PD (236 pg/mL). A group of *n* = 36 primarily non-demented patients with PD and PDD was clinically followed for up to two years. A higher baseline α-synuclein was associated with increases in Hoehn and Yahr classifications (*p* = 0.019) and Beck Depression Inventory scores (*p* < 0.001) as well as worse performance in Trail Making Test A (*p* = 0.017), Trail Making Test B (*p* = 0.043), and the Boston Naming Test (*p* = 0.002) at follow-up. Surprisingly, higher levels were associated with a better performance in semantic verbal fluency tests (*p* = 0.046). In summary, CSF α-synuclein may be a potential prognostic marker for disease progression, affective symptoms, and executive cognitive function in PD. Larger-scaled studies have to validate these findings and the discordant results for single cognitive tests in this exploratory investigation.

## 1. Introduction

Parkinson’s disease (PD) is a neurodegenerative disease caused by the demise of dopaminergic neurons in the substantia nigra. The exact etiology or the detailed pathogenesis is not yet fully clarified, but it seems to be an interplay of cellular and external environmental factors [1]. The prevalence of PD has increased dramatically over the years, affecting over six million people worldwide, most likely due to a longer duration of disease and better associated treatment options, and an increased number in the elderly population [2]. Today, PD, Lewy body dementia (DLB), and multiple system atrophy are grouped together as synucleinopathies, diseases that share Lewy bodies as a pathological commonality, in which α-synuclein is the major component [3]. Despite intensive research on α-synuclein and cellular inclusions, it is still unclear whether they are deleterious, nonfunctional, or even protective [4]. As summarized in a review by Lashuel et al. in 2013, α-synuclein has a physiological function in transmitter release in the synapse of neurons and is, thus, an important factor in neuronal plasticity. The production, function, and degradation of the protein is a regulated process that can lead to increased levels of the protein or oligomer formation when imbalanced, which could lead to the development of synucleinopathies [5]. It is thought that the pathologically occurring misfolded α-synuclein fibrils may spread from one neuron to the next, similar to prion proteins, and thereby promote disease progression [6,7].

In addition to the primary dementia syndrome with Lewy body pathology (DLB), the risk of developing dementia is increased six-fold in PD patients [8] and dementia in PD (PDD) is commonly distinguished from DLB by an onset of Parkinsonism and dementia after more than one year (PDD) [9]. Although these entities may be considered as subtypes in the spectrum of Lewy body diseases, a clearer pathophysiological and etiological distinction is needed [10]. Furthermore, the clinical differentiation of etiologies is complicated by the fact that, in addition to synucleinopathies (PD, DLB, and multiple system atrophy), other diseases, such as tauopathies (progressive supranuclear palsy and corticobasal degeneration) [11] and neurovascular diseases, may also present the clinical characteristics of Parkinsonism. As with other neurodegenerative and neurovascular diseases, the co-morbidity of distinct pathophysiologies may be frequent but hardly identifiable from a clinical point of view [12,13]. Several imaging techniques have been established (e.g., MRI, PET, SPECT, and sonography) to aid the differential diagnosis and to identify protein characteristics or metabolic alterations in vivo (MR-spectroscopy) [14]. However, to date, no biomarkers in the blood or cerebrospinal fluid have been established that can be used to confirm the diagnosis or predict the severity or mildness of the disease in PD or DLB. However, CSF α-synuclein levels were reported to be decreased in PD patients compared to controls [15], analogous to beta-amyloid 1–42 in Alzheimer’s disease. 

The aims of this work include the evaluation of the differences between CSF α-synuclein levels in patients with Lewy body diseases with and without dementia. To investigate the potential of CSF α-synuclein as a prognostic marker in PD, we explore the associations between the baseline concentrations and the progression of cognitive and motor symptoms during a two-year follow-up period in primarily non-demented PD and PDD patients. 

## 2. Materials and Methods

### 2.1. Study Cohort

Study patients were recruited for a prospective longitudinal study on cognitive decline in Parkinson’s disease and atypical Parkinson syndromes (PARKA) between 2009 and 2019 (*n* = 181). Follow-up visitations with longitudinal evaluations of clinical parameters were performed at 6, 12, and 24 months after baseline. For the presented study, patients with PD (*n* = 28), PDD (*n* = 8), and DLB (*n* = 5) were selected based on the availability of baseline CSF samples and data from two years of follow-up investigation. The International Movement Disorder Society criteria [16] were used to diagnose PD. The diagnosis of DLB as well as the discrimination of DLB and PDD (in PDD, dementia occurred > one year after the onset of Parkinsonism) was based on the fourth report the DLB Consortium [9]. The PDD group included *n* = 2 patients with mild cognitive impairment who were not considered separately. 

### 2.2. Clinical Test Scales, CSF Sampling, and α-Synuclein Measurement

The clinical parameters were evaluated through standardized questionnaires and rating scales by trained physicians and neuropsychologists, respectively. The neuropsychological tests included the simplified Beck Depression Inventory (BDI-V, cut-off ≥ 36 points indicates a high probability of a major depression) [17] and the Consortium to Establish a Registry for Alzheimer’s Disease (CERAD)-Plus test battery [18]. For the evaluation of motor symptoms and general disease stage, the third part (motor symptoms) of the Unified Parkinson’s Disease Rating Scale (UPDRS) [19] and the Hoehn and Yahr (H + Y) classification [20] were applied.

CSF samples were obtained, processed, and pseudonymized according to standard conditions. Subsequently, the samples were stored at −80 °C. No thawing or other use occurred until measurement. Prior to measurement, possible blood contamination was determined using Hemastrix strips (Siemens). All samples containing more than 25 erythrocytes/mm^3^ or hemoglobin were excluded from the measurement to avoid false-positive results (Llorens et al., 2018). The measurement of α-synuclein in human CSF was performed using an electrochemiluminescence immunoassay from Meso Scale Discovery MESO SCALE DIAGNOSTICS, LLC., Rockville, MD, USA, catalog No. K151TGD). The protocol for measurement was previously applied and described by our research group [21,22].

### 2.3. Statistical Methods

The differences in α-synuclein levels and clinical data between PD and Lewy body diseases with dementia (LBDD, PDD + DLB) were analyzed with two-tailed Mann–Whitney U tests. Mixed linear regression models were applied to investigate the influence of baseline α-synuclein as a continuous non-normally distributed variable regarding fixed and changing factors as well as different time points [23] at baseline, 6 months, 12 months, and 24 months in relation to α-synuclein. Estimates (coefficient of the equation) and *p*-values from these calculations refer to the correlation of baseline clinical item scores and CSF α-synuclein (*αSyn*), changes in the item score over time (*time*), and to the association between CSF α-synuclein and changes in an item score over time (*αSyn:time*). The results from the CERAD test items were transformed into Z-scores [24] that refer to an individual factor for the standard deviation (SD) above or below the norm, adjusted for age, sex, and education (e.g., MMSE z-score = −2.0 indicates that the MMSE score is two SD below the adjusted norm). 

We used SPSS^®^ and Microsoft^®^ Excel for the descriptive analyses and the creation of graphical items and RStudio© (RStudio PBC, Boston, MA, USA) version 1.1.463 (each test was analyzed with the function “lmer” from the package “lme4”) [25] for mixed linear regression models. A *p*-value of ≤ 0.05 was considered statistically significant. Because of the exploratory nature of the work, a *p*-value adjustment according to Bonferroni was omitted.

## 3. Results

### 3.1. Demographic Information and Baseline Data in PD, PDD, and DLB

**Figure 1:**
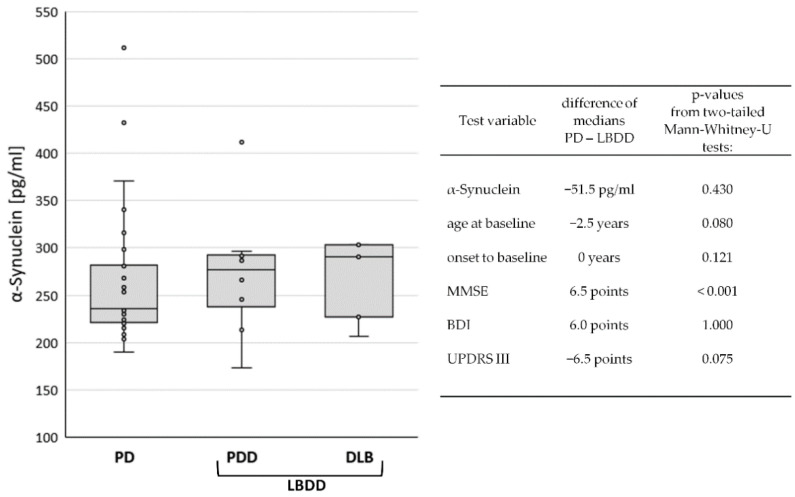
**Comparison of baseline data between diagnostic groups.** Box Plot of CSF α-synuclein levels in Parkinson’s disease (PD), Parkinson’s disease dementia (PDD), and dementia with Lewy bodies (DLB), left panel. In the right panel, differences in mean values and *p*-values from group comparisons between PD and Lewy body disease with dementia (LBDD) are displayed.

The study cohort (*n* = 41) consisted of patients with PD (*n* = 28), PDD (*n* = 8), and DLB (*n* = 5). PD patients without dementia (*n* = 28) were composed of 79% male (*n* = 22) and 21% female (*n* = 6) participants. In PDD (87.5%) and DLB (100%), the rate of male patients was even higher. The median ages at baseline were 69 (PD), 74 (PDD), and 71 (DLB) years. However, the time from disease onset to study baseline was only two years in PD and one year in DLB but was six years in PDD patients. Regarding the global cognitive status at baseline, the MMSE scores were naturally lower in PDD (24, IQR 6) and DLB (20, IQR 4) than in PD (29, IQR 2). Motor symptoms were also more severe in PDD (UPDRS III: 24, IQR 12) and DLB (UPDRS III: 23, IQR 7) than in PD (UPDRS 17, IQR 7). In contrast, the median BDI-V scores were higher in PD (28, IQR 21) than in PDD (23, IQR 36) and DLB (18, IQR 3), apparently indicating more signs of depression in the non-demented group. A summary of the data can be found in Table 1. The median CSF levels of α-synuclein were 236 pg/mL (IQR 61) in PD, 277 pg/mL (IQR 64) in PDD, and 291 pg/mL (IQR 76) in DLB (Figure 1). 

Due to the low case number in the demented diagnostic groups, group differences were only investigated between the non-demented PD patients (*n* = 28) and Lewy body diseases with dementia (LBDD = PDD plus DLB, *n* = 13). Although the median CSF levels of α-synuclein in PD (231 pg/mL) were lower (−51.5 pg/mL) than in LBDD (287 pg/mL), the difference was not statistically significant (*p* = 0.430). Similarly, the differences in age, disease duration, and BDI and UPDRS scores were not significant. Only the MMSE score was significantly lower in the LBDD group (*p* > 0.001), see Figure 1.

### 3.2. Association of CSF α-Synuclein Levels and Motor Symptom Progression in PD

For follow-up investigations, only patients with an initial diagnosis of PD at onset and no dementia until at least one year after were considered. This group consisted of *n* = 36 patients at baseline. The clinical disease stage showed a median of 1.5 on the Hoehn and Yahr scale, motor symptoms were rated a median of 18 in the UPDRS III, and affective symptoms were displayed a median of 23 points in the BDI. However, some data were lost to follow-up (Table 2). Especially after 6 months, some of the items could not be obtained because in most of the patients only a telephone visit was performed at 6 months. In other patients, the study was discontinued for personal or illness reasons or death. 

**Figure 2:**
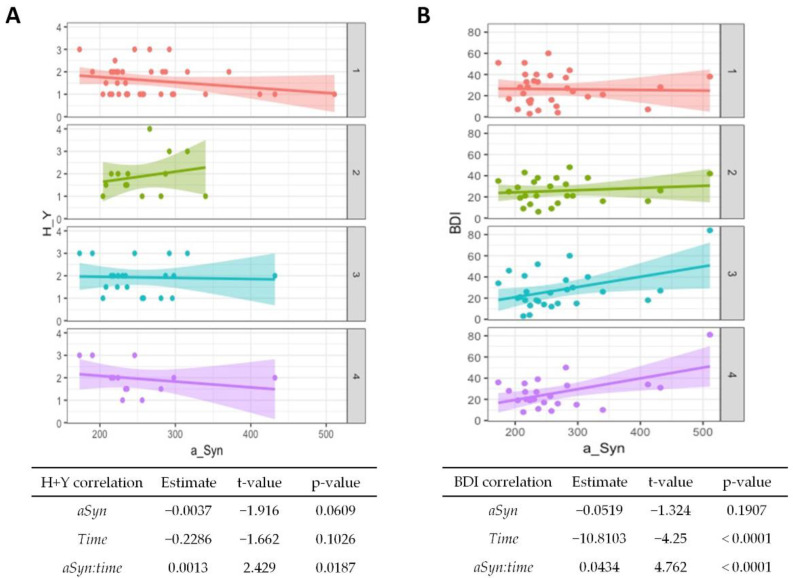
**Association of α-synuclein with disease progression and affective symptoms in PD.** Correlations from mixed linear regression are displayed by scatter plots with regression lines at baseline (1, red plot), 6 months (2, green plot), 12 months (3, turquoise plot), and 24 months (4, purple plot). Estimates, t-values, and *p*-values refer to the correlation of Hoehn and Yahr (H + Y) stage (**A**) and Beck Depression Inventory (BDI) scores (**B**) with α-synuclein (*αSyn*), changes in the stage/score over time (*time*), and to the correlation of baseline CSF α-synuclein with changes in stage/score over time (*αSyn:time*).

In the mixed linear regression model, the significance of changes in follow-up investigations was estimated independent of the lost follow-up data. Here, only the BDI showed significant alterations and decreased over time (*p* < 0.001), whereas the Hoehn and Yahr and UPDRS scores showed no significant correlation with time progression. The results are given in Figure 2A,B (correlation with *time*) and Supplementary Table S1.

The CSF α-synuclein levels showed no significant association with clinical scales at baseline, but a higher value at baseline was associated with an increase in the Hoehn and Yahr stage with passing time (*p* = 0.02, Figure 2A). Higher baseline values were also associated with poorer BDI results over time (*p* < 0.001, Figure 2B), although the overall test score generally improved.

### 3.3. Association of CSF α-Synuclein Levels and Cognitive Decline in PD

**Figure 3:**
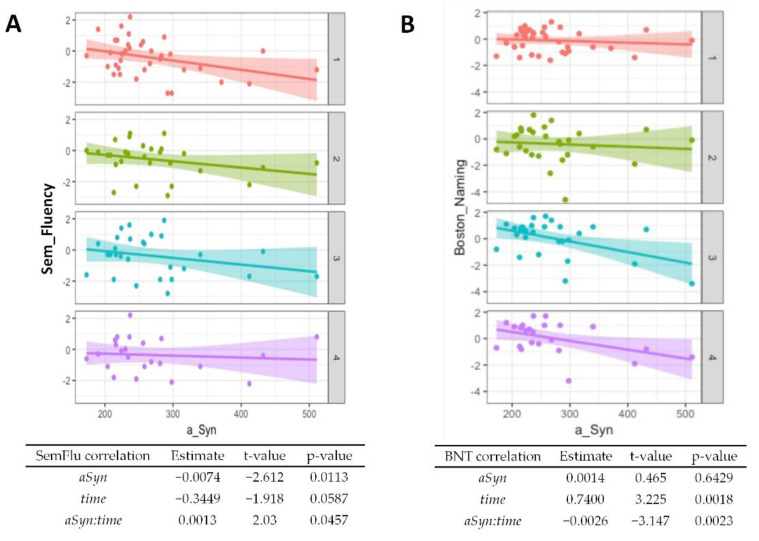

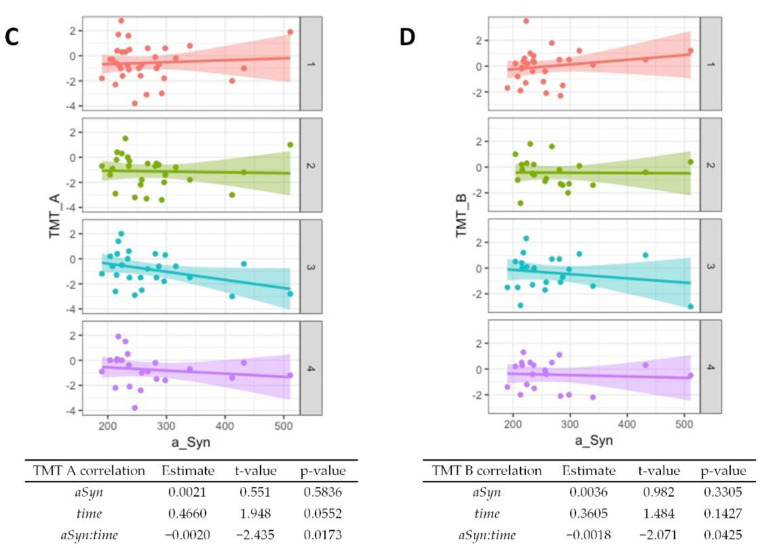
**Association of α-synuclein with cognitive test performance.** Correlations from mixed linear regression are displayed through scatter plots with regression lines at baseline (1, red plot), 6 months (2, green plot), 12 months (3, turquoise plot), and 24 months (4, purple plot). Estimates, t-values, and *p*-values refer to the correlation of results from the Boston Naming Test (BNT) (**A**), Semantic verbal fluency (SemFlu) (**B**), Trail Making Test A (TMT A) (**C**), and Trail Making Test B (TMT B) (**D**) with α-synuclein (*αSyn*), changes over time (*time*), and to the correlation of baseline CSF α-synuclein with changes in stage/score over time (*αSyn:time*).

Regarding baseline results from the CERAD cognitive test battery, worse performances of semantic verbal fluency (*p* = 0.01, Figure 3A) and word list savings (*p* = 0.04, Supplementary Table S1) were associated with higher α-synuclein levels. The performance in the Boston Naming Test generally improved over time (*p* = 0.002, Figure 3B) but higher α-synuclein at baseline was associated with worse performance at follow-up (*p* = 0.002). An association of higher baseline α-synuclein and worse performance at follow-ups was also observed for the Trail Making Test A (Figure 3C, *p* = 0.02) and the Trail Making Test B (Figure 3D, *p* = 0.04). A summary of the CERAD item z-scores at baseline and follow-ups is given in Supplementary Table S2.

## 4. Discussion

The model that explains the pathogenesis of PD by a deposition of misfolded α-synuclein suggests that CSF α-synuclein may serve as a potential diagnostic and prognostic marker. However, the literature regarding α-synuclein and cognition is sparse and heterogeneous. There is neither an established uniform cognitive test battery nor a uniform measurement of CSF α-synuclein. Our results indicate that relatively high levels may indicate a faster disease progression and a deterioration of cognition (Figure 2 and Figure 3). It might also be a predictor of the occurrence of depressed mood (Figure 3). On the other hand, CSF α-synuclein does not seem to discriminate PD from dementia syndromes with Lewy bodies (Figure 1).

### 4.1. CSF α-Synuclein in the Diagnostic Groups

The association of α-synuclein with PD has been a frequently studied topic in recent years. It has been studied in CSF, serum, saliva, and other tissues [26]. In previous studies, lower levels were seen in PD compared to controls [15,27,28], whereas patients with Lewy body diseases with dementia (PDD and DLB) showed higher levels than those with multiple system atrophy and PD [15,26]. However, meta-analyses showed that overall results remained highly heterogeneous [27,28]. We could not obtain a control group for our investigation, but a previous study from our group has measured a mean CSF α-synuclein level of 297 pg/mL in a control group using the same methods as in the presented work [29], which is apparently higher than in the PD group of the current study. A possible explanation for slightly higher levels in the demented patients of the cohort might be that PD patients in early stages may have lower levels than in later stages [29,30]. The formation of Lewy bodies may be a reason for the decreased amount of measured α-synuclein in early PD, but an increased release of the protein due to progressive neurodegeneration may lead to higher levels in the presence of dementia. In this respect, high CSF α-synuclein may be a well-suited candidate for being a stage-related and prognostic biomarker in PD.

### 4.2. Associations with Symptoms and Disease Progression

Although our study did not show a significant association between the biomarker and the baseline H + Y scale, in-line with previous studies [31,32], higher levels were significantly associated with a higher H + Y scale at follow-up (*p* = 0.02). With respect to the UPDRS III as a score for motor symptom severity, this work found no significant associations, which is also consistent with the current literature [30,32,33]. Concerning cognitive decline, our study suggests that CSF α-synuclein may be a prognostic marker for cognitive decline, especially in terms of executive functions. Although many items of the CERAD-Plus battery showed even slight improvement over time, possibly due to a learning effect, higher biomarker levels were associated with a worse performance on the Boston Naming test (*p* = 0.002), Trail Making Test A (0.017), and B (0.043). No other longitudinal studies using the Boston Naming test were found in the literature, but regarding the association of the deterioration of cognitive speed and elevated α-synuclein, another study showed similar results [34]. Here, however, the AQT (A Quick Test for Cognitive Speed) was used. A decline on other cognitive scales was not observed in this study. One explanation may be that, e.g., memory impairment is not a prominent feature in PD-related dementia [35], whereas data on the associations between CSF α-synuclein and visuoconstructional abilities are inconclusive [36,37]. Another explanation, also introducing a major limitation of this study, is the relatively slow disease progression of PD. It takes approximately two years to progress from H + Y stage one to two [38], which argues for investigations in a longitudinal design of at least four years. The development of dementia may take ten years or more in PD [39] and the two-year period observed here might be too short to draw a maximum conclusion from the cognitive data. However, in this patient collective, even discrete deficits could give an important indication of the cognitive development in PD due to the extensive neuropsychological testing. Regarding semantic fluency, we even observed a significant improvement in the test performance in relation to a higher baseline CSF α-synuclein (*p* = 0.046). Here, a learning effect may not fully explain the relation to increased α-synuclein, which is particularly interesting because other significant results showed an inverse relationship. On the other hand, semantic fluency showed a weak significance and a rather low estimate value compared to the other subtests. The current literature does not show any utility for α-synuclein as a prognostic parameter in PD [40]. Depression accompanies approximately 38% of PD patients and appears to be more common in subcortical dementias than in primarily cortical dementias, such as Alzheimer’s disease [41]. The evaluation of the patient’s mood by the BDI-V complemented the overall cognitive picture in our study cohort. Here, a higher baseline CSF α-synuclein was strongly associated with worse depressive symptoms at follow-up (*p* < 0.001), although BDI-V scores generally improved over time (*p* < 0.001). Another study also reported an improvement in depression scale scores over a two-year period in PD patients but did not observe a significant correlation with CSF α-synuclein [40]. Depression and cognitive impairment may not have a linear relationship over the PD course [42], but our results indicate a high potential for the biomarker as a predictor of depressive symptoms.

### 4.3. Study Limitations

At this point, we emphasize that the suitability of the investigated biomarker could only be assessed to a limited extent due to the explorative approach with multiple comparisons, a rather small sample size, and the absence of a control group, which are the major limitations of the study. As the presented work is a single-center study, the results should be validated independently using larger cohorts. Our study participants were diagnosed by clinical criteria, which may not be able to fully exclude co-pathologies or misdiagnoses. A neuropathology-confirmed study cohort is desirable but will be hard to realize within a prospective study design. Similarly, serial CSF samples would be helpful to analyze the stage-related evolution of the biomarker in an individual, but the serial application of an invasive procedure such as the lumbar puncture without any beneficial effect for the potentially demented study participant is ethically questionable and hard to realize. Peripheral fluid biomarkers may be the only way to acquire large-scaled longitudinal data in the future. To optimize the results of future studies, separate groups of demented and non-demented PD patients as well as healthy controls should be included in a longitudinal design. It would also be desirable to choose a uniform α-synuclein measurement method. In addition, efforts should be undertaken to agree on neuropsychological testing in favor of better comparability. The CERAD-Plus test battery seems to be a suitable starting point for this and forms a major strength of this work because of its general distribution, available normative results, and a coverage of all important cognitive domains. In the future, total CERAD scores, such as those that have been tested in Alzheimer’s patients [43], or specific subscores that have been suggested for PD [35], should be investigated.

### 4.4. Conclusions and Future Perspectives

In conclusion, α-synuclein may be a promising biomarker for disease progression, cognitive decline, and depressive mood in PD. However, the clinical utility needs to be assessed in larger studies, and the ostensible discrepancy that CSF α-synuclein was reported to be lower in PD compared to controls, whereas higher levels may be associated with faster PD progression, has to be clarified. Co-morbidities that also contribute to cognitive decline, such as other neurodegenerative or even primarily non-neurological diseases, have to be considered. The role of α-synuclein is not exhausted in being the major component of Lewy bodies. Its function as an indicator for synaptic damage and potential involvement in tumor genesis [44] as well as the interplay between the mentioned mechanisms require further investigation. Comparative and integrative investigations of α-synuclein and other biomarker candidates, such as neurofilament light chain, which was reported to be elevated in DLB compared to non-demented PD patients in CSF and blood plasma [45,46] and to be associated with symptom progression [47], may be able to improve the prognostic potential of fluid biomarker analyses.

To this end, the CERAD-Plus test battery may be a suitable cognitive measurement tool in conjunction with a unified α-synuclein measurement method. Advances in diagnostics and a better understanding of the disease ultimately serve the exploration of new therapeutic options and, thus, an improvement in the daily lives and quality of life of affected individuals.

## Figures and Tables

**Table 1:** Baseline data and α-synuclein in the diagnostic groups.

	PD	PDD	DLB	LBDD

*n*	28	8	5	13
CSF α-synuclein: median (IQR) (pg/mL)	236 (61)	277 (64)	291 (76)	287 (73)
sex: female/male	6/22	1/7	0/5	1/12
age at sampling: median (IQR) (years)	69 (16)	74 (8)	70 (6)	71 (7)
onset to baseline: median (IQR) (years)	2 (3)	6 (10)	1 (2)	2 (6)
MMSE: median (IQR) (score)	29 (2)	24 (6)	20 (4)	22 (6)
BDI: median (IQR) (score)	28 (21)	23 (36)	18 (3)	22 (18)
UPDRS III: median (IQR) (score)	17 (7)	24 (12)	23 (7)	24 (12)

PD: Parkinson’s disease; PDD: Parkinson’s disease dementia; DLB: dementia with Lewy bodies; LBDD: Lewy body disease with dementia (DLB + PDD); IQR: interquartile range; MMSE: Mini Mental Status Examination; BDI: Beck Depression Inventory; UPDRS III: motor symptom score of the Unified Parkinson’s Disease Rating Scale.

**Table 2:** Disease stage, motor symptoms, and depression scales at baseline and follow-up.

PD + PDD	Hoehn + Yahr Stage		UPDRS Score		BDI Score	
	*n*	Median (IQR)	*n*	Median (IQR)	*n*	Median (IQR)

Baseline	36	1.5 (1)	36	18 (10)	31	23 (22.25)
6 months	14	1.75 (1)	4	17 (14)	25	20 (19)
12 months	21	2 (0.5)	17	19 (5)	27	19.5 (19.5)
24 months	13	2 (0.5)	19	20 (9)	23	17 (17)

PD: Parkinson’s disease; PDD: Parkinson’s disease dementia; IQR: interquartile range; BDI: Beck Depression Inventory; UPDRS III: motor symptom score of the Unified Parkinson’s Disease Rating Scale.

## Data Availability

The data presented in this study are available on request from the corresponding author.

## Supplementary Materials

The following supporting information can be downloaded at: https://www.mdpi.com/article/10.3390/diagnostics12051259/s1, Table S1: Results from linear regression models in PD and PDD; Table S2: CERAD item z-scores at baseline and over time in PD and PDD.

## References

[1] Kalia L.V., Lang A.E. (2015). Parkinson’s disease. Lancet.

[2] GBD 2016 Parkinson’s Disease Collaborators (2018). Global, regional, and national burden of Parkinson’s disease, 1990–2016: A systematic analysis for the Global Burden of Disease Study 2016. Lancet Neurol..

[3] Goedert M., Spillantini M.G., Del Tredici K., Braak H. (2013). 100 years of Lewy pathology. Nat. Rev. Neurol..

[4] Brás I.C., Dominguez-Meijide A., Gerhardt E., Koss D., Lázaro D.F., Santos P.I., Vasili E., Xylaki M., Outeiro T.F. (2020). Synucleinopathies: Where we are and where we need to go. J. Neurochem..

[5] Lashuel H.A., Overk C.R., Oueslati A., Masliah E. (2013). The many faces of α-synuclein: From structure and toxicity to therapeutic target. Nat. Rev. Neurosci..

[6] Olanow C.W., Brundin P. (2013). Parkinson’s disease and alpha synuclein: Is Parkinson’s disease a prion-like disorder?. Mov. Disord..

[7] Woerman A.L., Stöhr J., Aoyagi A., Rampersaud R., Krejciova Z., Watts J.C., Ohyama T., Patel S., Widjaja K., Oehler A. (2015). Propagation of prions causing synucleinopathies in cultured cells. Proc. Natl. Acad. Sci. USA.

[8] Aarsland D., Andersen K., Larsen J.P., Lolk A., Nielsen H., Kragh-Sørensen P. (2001). Risk of dementia in Parkinson’s disease: A community-based, prospective study. Neurology.

[9] McKeith I.G., Boeve B.F., Dickson D.W., Halliday G., Taylor J.-P., Weintraub D., Aarsland D., Galvin J., Attems J., Ballard C.G. (2017). Diagnosis and management of dementia with Lewy bodies: Fourth consensus report of the DLB Consortium. Neurology.

[10] Jellinger K.A., Korczyn A.D. (2018). Are dementia with Lewy bodies and Parkinson’s disease dementia the same disease?. BMC Med..

[11] Ludolph A.C., Kassubek J., Landwehrmeyer B.G., Mandelkow E., Mandelkow E.M., Burn D.J., Caparros-Lefebvre D., Frey K.A., de Yebenes J.G., Gasser T. (2009). Reisensburg Working Group for Tauopathies with Parkinsonism. Tauopathies with parkinsonism: Clinical spectrum, neuropathologic basis, biological markers, and treatment options. Eur. J. Neurol..

[12] Mooney T., Tampiyappa A., Robertson T., Grimley R., Burke C., Ng K., Patrikios P. (2011). Dual pathology of corticobasal degeneration and Parkinson’s disease in a patient with clinical features of progressive supranuclear palsy. Neurol. India.

[13] Alster P., Madetko N., Koziorowski D., Friedman A. (2020). Progressive Supranuclear Palsy-Parkinsonism Predominant (PSP-P)—A Clinical Challenge at the Boundaries of PSP and Parkinson’s Disease (PD). Front. Neurol..

[14] Saeed U., Lang A.E., Masellis M. (2020). Neuroimaging Advances in Parkinson’s Disease and Atypical Parkinsonian Syndromes. Front. Neurol..

[15] Mollenhauer B., Locascio J.J., Schulz-Schaeffer W., Sixel-Döring F., Trenkwalder C., Schlossmacher M.G. (2011). α-Synuclein and tau concentrations in cerebrospinal fluid of patients presenting with parkinsonism: A cohort study. Lancet Neurol..

[16] Postuma R.B., Berg D., Stern M., Poewe W., Olanow C.W., Oertel W., Obeso J., Marek K., Litvan I., Lang A.E. (2015). MDS clinical diagnostic criteria for Parkinson’s disease. Mov. Disord..

[17] Schmitt M., Maes J. (2000). Vorschlag zur Vereinfachung des Beck-Depressions-Inventars (BDI). Diagnostica.

[18] Schmid N.S., Ehrensperger M.M., Berres M., Beck I.R., Monsch A.U. (2014). The Extension of the German CERAD Neuropsychological Assessment Battery with Tests Assessing Subcortical, Executive and Frontal Functions Improves Accuracy in Dementia Diagnosis. Dement. Geriatr. Cogn. Dis. Extra.

[19] Fahn S., Elton R.L., Fahn S., Marsden C.D., Goldstein M., Stoker T.B., Barker R.A., Members of the UPDRS Development Committee (1987). Unified Parkinson’s disease rating scale IN. Recent Developments in Parkinson’s Disease.

[20] Goetz C.G., Poewe W., Rascol O., Sampaio C., Stebbins G.T., Counsell C., Giladi N., Holloway R.G., Moore C.G., Wenning G.K. (2004). Movement Disorder Society Task Force report on the Hoehn and Yahr staging scale: Status and recommendations. Mov. Disord..

[21] Llorens F., Kruse N., Schmitz M., Shafiq M., da Cunha J.E., Gotzman N., Zafar S., Thüne K., De Oliveira J.R.M., Mollenhauer B. (2015). Quantification of CSF biomarkers using an electrochemiluminescence-based detection system in the differential diagnosis of A.D and sCJD. J. Neurol..

[22] Llorens F., Kruse N., Karch A., Schmitz M., Zafar S., Gotzmann N., Sun T., Köchy S., Knipper T., Cramm M. (2018). Validation of α-Synuclein as a CSF Biomarker for Sporadic Creutzfeldt-Jakob Disease. Mol. Neurobiol..

[23] Luck T., Riedel-Heller S.G., Wiese B., Stein J., Weyerer S., Werle J., Kaduszkiewicz H., Wagner M., Mosch E. (2009). CERAD-NP-Testbatterie: Alters-, geschlechts- und bildungsspezifische Normen ausgewählter Subtests. Ergebnisse der German Study on Ageing, Cognition and Dementia in Primary Care Patients (AgeCoDe) [CERAD-NP battery: Age-, gender- and education-specific reference values for selected subtests. Results of the German Study on Ageing, Cognition and Dementia in Primary Care Patients (AgeCoDe)]. Z. Gerontol. Geriatr..

[24] Verbeke G., Molenberghs G. (2000). Linear Mixed Models for Longitudinal Data.

[25] Bates D., Mächler M., Bolker B., Walker S. (2015). Fitting Linear Mixed-Effects Models Using lme4. J. Stat. Soft..

[26] Fayyad M., Salim S., Majbour N., Erskine D., Stoops E., Mollenhauer B., El-Agnaf O.M.A. (2019). Parkinson’s disease biomarkers based on α-synuclein. J. Neurochem..

[27] Gao L., Tang H., Nie K., Wang L., Zhao J., Gan R., Huang J., Zhu R., Feng S., Duan Z. (2015). Cerebrospinal fluid alpha-synuclein as a biomarker for Parkinson’s disease diagnosis: A systematic review and meta-analysis. Int. J. Neurosci..

[28] Simonsen A.H., Kuiperij B., El-Agnaf O.M.A., Engelborghs S., Herukka S.-K., Parnetti L., Rektorova I., Vanmechelen E., Kapaki E., Verbeek M. (2016). The utility of α-synuclein as biofluid marker in neurodegenerative diseases: A systematic review of the literature. Biomark. Med..

[29] Llorens F., Schmitz M., Varges D., Kruse N., Gotzmann N., Gmitterová K., Mollenhauer B., Zerr I. (2016). Cerebrospinal α-synuclein in α-synuclein aggregation disorders: Tau/α-synuclein ratio as potential biomarker for dementia with Lewy bodies. J. Neurol..

[30] Mollenhauer B., Caspell-Garcia C.J., Coffey C.S., Taylor P., Singleton A., Shaw L.M., Trojanowski J.Q., Frasier M., Simuni T., Iranzo A. (2019). Longitudinal analyses of cerebrospinal fluid α-Synuclein in prodromal and early Parkinson’s disease. Mov. Disord..

[31] Hall S., Öhrfelt A., Constantinescu R., Andreasson U., Surova Y., Bostrom F., Nilsson C., Widner H., Decraemer H., Nägga K. (2012). Accuracy of a panel of 5 cerebrospinal fluid biomarkers in the differential diagnosis of patients with dementia and/or parkinsonian disorders. Arch. Neurol..

[32] Van Dijk K.D., Bidinosti M., Weiss A., Raijmakers P., Berendse H.W., Van De Berg W.D. (2014). Reduced α-synuclein levels in cerebrospinal fluid in Parkinson’s disease are unrelated to clinical and imaging measures of disease severity. Eur. J. Neurol..

[33] Hall S., Surova Y., Öhrfelt A., Blennow K., Zetterberg H., Hansson O., Swedish BioFINDER Study (2016). Longitudinal Measurements of Cerebrospinal Fluid Biomarkers in Parkinson’s Disease. Mov. Disord..

[34] Hall S., Surova Y., Öhrfelt A., Zetterberg H., Lindqvist D., Hansson O. (2015). CSF biomarkers and clinical progression of Parkinson disease. Neurology.

[35] Lillig R., Ophey A., Schulz J.B., Reetz K., Wojtala J., Storch A., Liepelt-Scarfone I., Becker S., Berg D., Balzer-Geldsetzer M. (2021). A new CERAD total score with equally weighted z-scores and additional executive and non-amnestic “CERAD-Plus” tests enhances cognitive diagnosis in patients with Parkinson’s disease: Evidence from the LANDSCAPE study. Parkinsonism Relat. Disord..

[36] Compta Y., Valente T., Saura J., Segura B., Iranzo Á., Serradell M., Junque C., Tolosa E., Valldeoriola F., Muñoz E. (2015). Correlates of cerebrospinal fluid levels of oligomeric- and total-α-synuclein in premotor, motor and dementia stages of Parkinson’s disease. J. Neurol..

[37] Stav A.L., Aarsland D., Johansen K.K., Hessen E., Auning E., Fladby T. (2015). Amyloid-β and α-synuclein cerebrospinal fluid biomarkers and cognition in early Parkinson’s disease. Parkinsonism Relat. Disord..

[38] Zhao Y.J., Wee H.L., Chan Y.-H., Seah S.H., Au W.L., Lau P.N., Pica E.C., Li S.C., Luo N., Tan L.C. (2010). Progression of Parkinson’s disease as evaluated by Hoehn and Yahr stage transition times. Mov. Disord..

[39] Aarsland D., Kurz M.W. (2010). The epidemiology of dementia associated with Parkinson’s disease. Brain Pathol..

[40] Mollenhauer B., Zimmermann J., Sixel-Döring F., Focke N.K., Wicke T., Ebentheuer J., Schaumburg M., Lang E., Trautmann E., Zetterberg H. (2016). Monitoring of 30 marker candidates in early Parkinson disease as progression markers. Neurology.

[41] Turner M.A., Moran N.F., Kopelman M.D. (2002). Subcortical dementia. Br. J. Psychiatry.

[42] Schroeders U., Zimmermann J., Wicke T., Schaumburg M., Lang E., Trenkwalder C., Mollenhauer B. (2022). Dynamic interplay of cognitive functioning and depressive symptoms in patients with Parkinson’s disease. Neuropsychology.

[43] Rossetti H.C., Munro Cullum C., Hynan L.S., Lacritz L.H. (2010). The CERAD Neuropsychologic Battery Total Score and the Progression of Alzheimer Disease. Alzheimer. Dis. Assoc. Disord..

[44] Ejma M., Madetko N., Brzecka A., Guranski K., Alster P., Misiuk-Hojło M., Somasundaram S.G., Kirkland C.E., Aliev G. (2020). The Links between Parkinson’s Disease and Cancer. Biomedicines.

[45] Schmitz M., Villar-Piqué A., Llorens F., Gmitterová K., Hermann P., Varges D., Zafar S., Lingor P., Vanderstichele H., Demeyer L. (2019). Cerebrospinal Fluid Total and Phosphorylated α-Synuclein in Patients with Creutzfeldt-Jakob Disease and Synucleinopathy. Mol. Neurobiol..

[46] Canaslan S., Schmitz M., Villar-Piqué A., Maass F., Gmitterová K., Varges D., Lingor P., Llorens F., Hermann P., Zerr I. (2021). Detection of Cerebrospinal Fluid Neurofilament Light Chain as a Marker for Alpha-Synucleinopathies. Front. Aging Neurosci..

[47] Mollenhauer B., Dakna M., Kruse N., Galasko D., Foroud T., Zetterberg H., Schade S., Gera R.G., Wang W., Gao F. (2020). Validation of Serum Neurofilament Light Chain as a Biomarker of Parkinson’s Disease Progression. Mov. Disord..

