# Supplementary material for: Baseline Cerebrospinal Fluid α-Synuclein in Parkinson’s Disease Is Associated with Disease Progression and Cognitive Decline
Source: Diagnostics (Basel). 2022 May 18;12(5):1259. doi: 10.3390/diagnostics12051259 (PMC9140902; doi:10.3390/diagnostics12051259)
Supplement: Supplementary file 1 [file diagnostics-12-01259-s001.zip › diagnostics-1661653-supplementary.pdf]

Table S1. Results from linear regression models in PD and PDD.

Clinical scores and CERAD test items	Estimate	t-value	p-value
**Hoehn and Yahr stage correlation**			
aSyn	-0.0037	-1.916	0.061
Time	-0.2286	-1.662	0.103
aSyn:time	0.0013	2.429	0.019*
UPDRS correlation			
aSyn	0.0145	0.551	0.583
Time	4.0400	1.263	0.113
aSyn:time	-0.0100	-1.372	0.179
**Beck Depression Inventory correlation**			
aSyn	-0.0519	-1.324	0.191
Time	-10.8103	-4.25	< 0.001***
aSyn:time	0.0433	4.762	< 0.001***
**Semantic verbal fluency correlation**			
aSyn	-0.0074	-2.612	0.011*
Time	-0.3449	-1.918	0.059
aSyn:time	0.0013	2.03	0.046*
**Boston Naming Test correlation**			
aSyn	0.0014	0.465	0.643
Time	0.7400	3.225	0.002**
aSyn:time	-0.0026	-3.147	0.002**
**Mini Mental Status correlation**			
aSyn	-0.0050	-1.033	0.305
Time	0.0503	0.158	0.874
aSyn:time	0.0002	0.153	0.879
**Word list correlation**			
aSyn	-0.0024	-0.608	0.545
Time	0.3653	1.617	0.110
aSyn:time	-0.0010	-1.285	0.202
**Word list recall correlation**			
aSyn	-0.0057	-1.779	0.079
Zeit	0.0352	0.149	0.882
aSyn:Zeit	0.0002	0.266	0.791

**Word list intrusions correlation**			
aSyn	0.0023	0.695	0.489
Zeit	0.0021	0.723	0.472
aSyn:Zeit	-0.0005	-0.474	0.637
**Word list savings correlation**			
aSyn	-0.0088	-2.047	0.043*
Zeit	-0.2108	-0.485	0.629
aSyn:Zeit	0.0017	1.059	0.293
**Word list discriminability**			
aSyn	-0.0022	-0.643	0.522
Zeit	0.3301	1.129	0.262
aSyn:Zeit	-0.0012	-1.180	0.241
**Visuoconstruction correlation**			
aSyn	0.0007	0.239	0.812
Zeit	0.0611	0.345	0.731
aSyn:Zeit	-0.0001	-0.175	0.862
**Figures recall correlation**			
aSyn	0.0014	0.351	0.726
Zeit	0.5105	1.548	0.126
aSyn:Zeit	-0.0016	-1.379	0.172
**Figures savings correlation**			
aSyn	-0.0012	-0.386	0.700
Zeit	0.1566	0.598	0.552
aSyn:Zeit	-0.0005	-0.530	0.598
**Phonematic fluency correlation**			
aSyn	-0.0018	-0.569	0.572
Zeit	-0.5830	-0.291	0.772
aSyn:Zeit	0.0004	0.061	0.951
**Trai Making Test A correlation**			
aSyn	0.0021	0.551	0.584
Zeit	0.4660	1.948	0.055
aSyn:Zeit	-0.0021	-2.435	0.017*
**Trai Making Test B correlation**			
aSyn	0.0036	0.982	0.331

Zeit	0.3605	1.484	0.143
aSyn:Zeit	-0.0018	-2.071	0.043*
**Trai Making Test B/A correlation**			
aSyn	-0.0019	-0.586	0.560
Zeit	-0.2096	-0.747	0.457
aSyn:Zeit	0.0008	0.797	0.428

Table S2. CERAD item z-scores at baseline and over time in PD and PDD.

CERAD items in PD (and PDD) patients	Baseline		6 months		12 months		24 months	
	n	z-score median (IQR)	n	z-score median (IQR)	n	z-score median (IQR)	n	z-score median (IQR)
Semantic fluency	*36*	-0.3 (1.6)	28	-0.3 (0.975)	28	-0.3 (2.175)	23	0.4 (1.6)
Semantic fluency*	23	-0.3 (1.65)						
Boston Naming Test	36	-0.1 (1.275)	28	-0.15 (1.65)	28	0.55 (1.25)	23	0.5 (1.7)
Boston Naming Test*	23	-0.1 (1.4)						
Mini Mental Status Examination	36	-1.35 (2.35)	28	-1.2 (1.9)	28	-0.45 (2.525)	23	-0.4 (1.7)
Mini Mental Status Examination*	23	-0.9 (2.35)						
Word list	*36*	0.15 (1.6)	28	0.45 (1.8)	28	0.55 (1.35)	23	0.8 (2.06)
Word list*	*23*	-0.3 (1.7)						
Word list recall	*36*	-0.25 (1.225)	28	-0.1 (1.775)	28	0 (0.825)	23	-0.1 (1.45)
Word list recall*	*23*	-0.1 (1.45)						
Word list intursions	36	0.4 (2.05)	28	0.6 (2.0)	28	0.55 (1.825)	23	0.6 (1.65)
Word list intursions*	23	0.4 (2.05)						
Word list savings	36	-0.6 (1.825)	28	-0.25 (2.225)	28	0.05 (1.15)	23	-0.1 (0.75)
Word list savings*	23	-0.3 (1.75)						
Word list discriminability	36	0.3 (2.025)	28	0.4 (1.525)	28	0.6 (1.625)	23	0.6 (1.7)
Word list discriminability*	23	0.6 (1.85)						
Visuoconstruction	36	0.25 (1.625)	28	0.35 (1.775)	28	0.4 (1.625)	23	0.6 (1.15)
Visuoconstruction*	23	0.4 (1.7)						
Figure recall	*36*	-0.8 (1.95)	28	-0.9 (2.05)	28	-0.05 (2.4)	23	-0.4 (2.15)
Figure recall*	*23*	-0.5 (.5)						
Figure savings	*36*	-0.6 (1.475)	28	-0.8 (1.65)	28	-0.2 (1.75)	23	-0.8 (1.85)
Figure savings*	*23*	-0.7 (1.65)						
Trail Making Test A	34	-0.7 (1.85)	26	-0.8 (2.3)	25	-0.7 (1.65)	22	-0.8 (1.475)
Trail Making Test A*	22	-0.45 (1.55)						
Trail Making Test B	29	0.2 (1.3)	21	-0.45 (1.45)	20	-0.1 (1.95)	20	-0.25 (1.775)
Trail Making Test B*	20	0.2 (0.9)						
Trail Making Test Ratio B/A	29	0.1 (1.2)	21	0.1 (1.1)	20	0.9 (1.4)	20	0.1 (1.35)
Trail Making Test Ratio B/A*	20	0.15 (1.75)						
Phonematic fluency	36	0 (1.5)	28	-0.3 (1.425)	28	-0.35 (1.625)	23	-0.3 (1.35)

Phonematic fluency*	23	0.3 (1.4)	

CERAD: Consortium to Establish a Registry for Alzheimer's Disease plus test battery; PD: Parkinson's Disease; PDD: Parkinson's Disease with Dementia; z-score: factor for test result above or below the norm, adjusted for age, sex, and education based on standard deviations (e.g. MMSE z-score = -2.0 indicates that the MMSE score is two-fold SD below the adjusted norm); IQR: Inter Quartile Range. *For this seperate display of item z-scores, only cases with available data at baseline and after 24 months are considered.
